# Lysosome stress response and mitochondria injury are the earliest detectable alteration in FSGS

**DOI:** 10.1038/s41598-025-22622-x

**Published:** 2025-10-13

**Authors:** Eileen Dahlke, Jessica Promnitz, Mairon Trujillo Miranda, Makhabbat Saudenova, Gèraldine Mollet, Franziska Theilig

**Affiliations:** 1 Institute of Anatomy, Christian-Albrechts-University Kiel, Otto-Hahn-Platz 8, 24118 Kiel, Germany; 2 Institut Imagine, Laboratory of Hereditary Kidney Diseases, INSERM UMR1163, Université Paris Cité, 75015 Paris, France

**Keywords:** Lysosome stress, Mitochondria injury, Podocyte, FSGS, Kidney, Kidney diseases

## Abstract

Focal segmental glomerulosclerosis (FSGS) is a histopathologic lesion caused by a dysfunction and loss of podocytes. Podocytes as postmitotic cells, rely on lysosomes to maintain their structural and functional integrity and on mitochondria to adapt their metabolic needs. The importance of lysosomes and mitochondria in glomerular diseases is widely accepted, their time-dependent involvement during pathogenic events of FSGS remain unknown. The inducible mouse model of podocyte-specific Nphs2 deletion allowing the study of the time-dependent sequence of pathogenic events. Earliest alterations were overserved at 5 days after FSGS induction and comprise a very low number of glomeruli with foot process effacement and tuft adhesions to the Bowmans capsule, a lysosome stress response, mitochondria injury and higher chemokine CXCL1 expression. At 9 days after FSGS induction, increased glomerular injury with additional mesangial proliferations, higher abundance of lysosomes, galectin-3 and mitochondria stress occurred. At 17 days after FSGS induction, severe glomerular injury with additional podocyte flattening, glomerular sclerosis and hyaline deposits together with ER stress were evident. In summary, lysosome stress response and mitochondria injury are the earliest events in FSGS development. Having a role in signaling and inflammation, both lysosomes and mitochondria may facilitate the initiating event of inflammation with subsequent chemokine CXCL1 action within the podocyte. Endoplasmic reticulum stress however, may maintain podocytes injury and contribute to the disease conservation of FSGS.

**Supplementary Information:**

The online version contains supplementary material available at 10.1038/s41598-025-22622-x.

## Introduction

Focal segmental glomerulosclerosis (FSGS) is a histopathologic lesion of renal glomeruli caused by a dysfunction and loss of podocytes due to various injurious stimuli. FSGS may describe a disease characterized by primary podocyte injury (primary FSGS) and also lesions developing secondarily to other types of chronic kidney disease (secondary FSGS)^1^. Pathogenic events include glomerular scar formation through capillary collapse, podocyte hypertrophy, podocyte foot process effacement with consecutive detachment and loss of podocyte into the urine, extracellular matrix accumulation and glomerular basement membrane thickening. Clinically, patients develop proteinuria and potentially kidney function decline as long-term outcome^1^.

Podocytes are terminally differentiated epithelial cells which are postmitotic^2^. To maintain their structural and functional integrity, podocytes need to renew their cellular components involving lysosomes as the major degradative compartment within the cell^3^. Lysosomes are now recognized as metabolic signaling hub and serve as a platform for physiological and pathological signaling that control podocyte homeostasis. One of the central signaling molecules on the lysosome surface is mechanistic target of rapamycin complex 1 (mTORC1), an important regulator of cell growth and autophagy with dynamic complex formations at lysosomes and other cell organelles^4^. Lysosomal mTORC1 substrates include transcription factor EB (TFEB), transcription factor 3 (TF3) and the regulator protein RagC^4^ for regulation of lysosomal biogenesis and autophagy^5^. Lysosome stress response, defined as a state where lysosome enter into upon external or internal stimuli^6^, utilizing the transcription response machinery to restore lysosome function. The important role of lysosomes in glomerular diseases have been shown^7^ and lysosome dysregulation is thought to be associated with podocyte injury and consecutive proteinuria^8^^,^^9^.

During the development and progression of FSGS mitochondria have also a critical role in the maintenance of podocyte homeostasis^10^. In mouse podocytes, mitochondrial respiration account for most of the cellular respiration which is mainly coupled to ATP^11^. An imbalance of mitochondrial homeostasis has been implicated in the progression of various glomerular diseases. Disturbances in mitochondrial dynamics lead to reduced ATP generation, calcium signaling and increased oxidative stress. Mitochondria are the major site of intracellular reactive oxygen species (ROS) generation which may trigger the opening of the mitochondrial permeability transition pore and lead to mitochondria dysfunction, organelle swelling and podocyte cell stress.

Although, the importance of lysosomes and mitochondria in glomerular diseases is widely accepted, the timeline of both organelle involvement in the pathogenic events of glomerular injury remains unknown. The inducible mouse model of podocyte-specific *Nphs2* deletion is used as excellent model to study podocyte injury and development of focal segmental glomerulosclerosis (FSGS) in a time-dependent sequence of pathogenic events^12^^,^^13^. Mice with inducible podocyte-specific *Nphs2* deletion develop albuminuria progressing to proteinuria, higher blood pressure and a decline in kidney at four weeks after disease induction. Aim of our study is to determine the timeline of pathogenic events during the development of FSGS and to integrate molecular with morphological glomerular alterations.

## Results

### Time-dependent assessment of glomerular injury during FSGS development

**Fig. 1:**
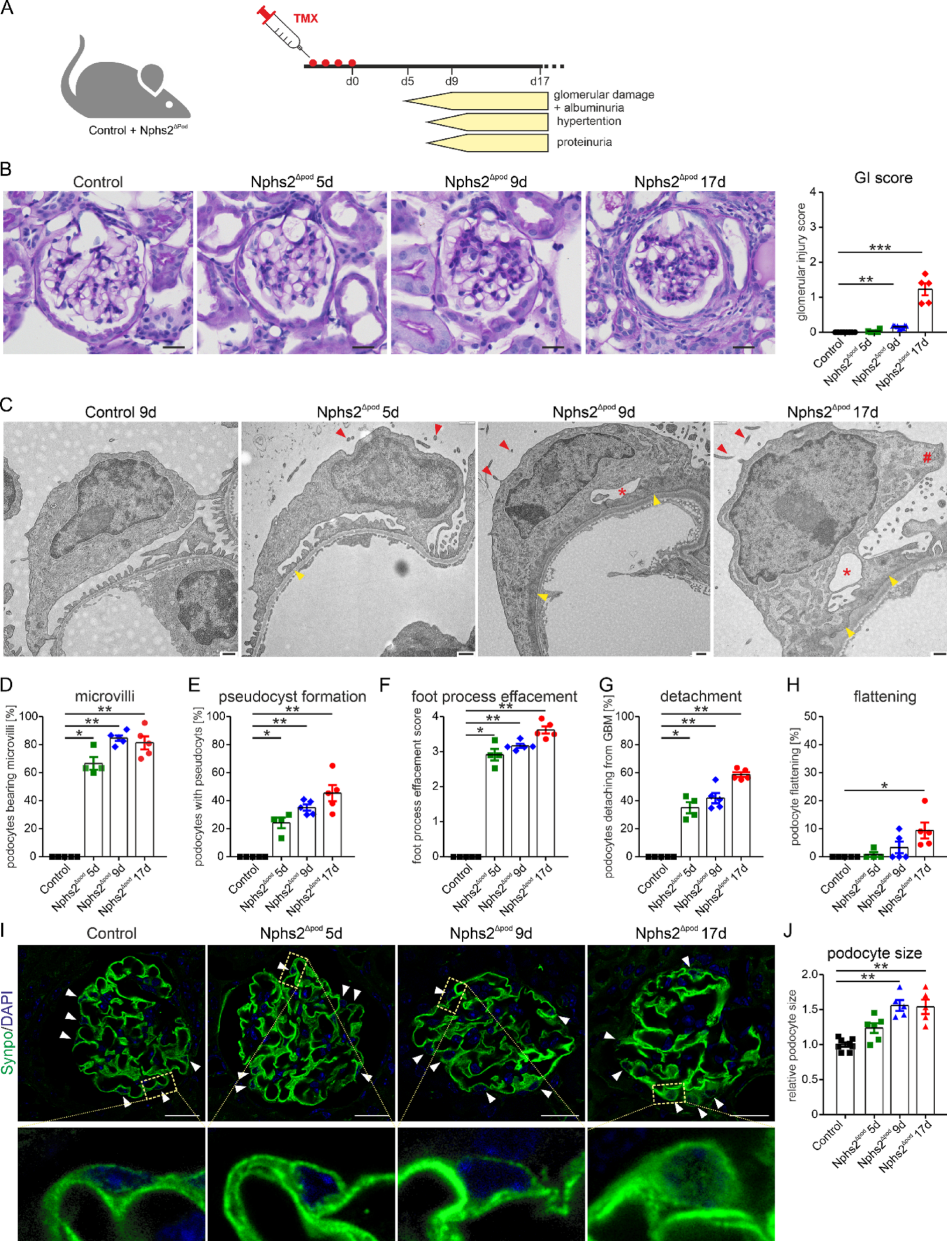
Morphological alterations in podocytes after FSGS induction in Nphs2^Δpod^. (**A**) Visualisation of the inducible transgenic mouse model and chronological onset of albuminuria, hypertension and proteinuria. (**B**) Representative PAS-stainings (left) and quantification (right) of the glomerular injury (GI) score in control and *Nphs2*^Δpod^ mice. Scale bar = 20 µm. n = 4–8 per group with > 40 glomeruli per n. **, *P* < 0.01 ; ***, *P* < 0.001. (**C**) Electron microscopy images of podocytes from control and *Nphs2*^Δpod^ mice. Red arrow heads point to apical microvilli and yellow arrow heads to enlarged foot processes; red asterisks mark pseudocysts and red hashtags podocyte detachment. Scale bar = 500 nm. (**D**–**H**) Quantification of podocyte microvilli (**D**), pseudocyst formation (**E**), foot process effacement (**F**), detachment from the glomerular basement membran (**G**) and flattened appearance (**H**). n = 4–5 per group with > 22 podocytes evaluated. *, *P* < 0.05; **, *P* < 0.01. (**I**) Represenative images obtained from glomeruli of each group stained against synaptopodin in green. White arrow heads point to individual podocytes. Scale bar = 20 µm. Higher magnification image is shown below. (**J**) Quantification of podocyte cell size. The mean of the control is set to 1 and compared to *Nphs2*^Δpod^. n = 5–8 per group with > 30 glomeruli evaluated. **, *P* < 0.01.

An established mouse model was used for the induction of the FSGS phenotype with a podocyte-specific deletion of Nphs2 (termed Nphs2^∆pod^)^12^^,^^13^, leading to progressive podocyte injury and altered renal functions (Fig. 1A). After FSGS induction albuminuria was starting at 5 days, hypertension and proteinuria at 9 days. Morphological assessment of glomerular injury of Periodic acid-Schiff (PAS)-stained renal section of *Nphs2*^∆pod^ revealed in comparison to control at 5 days after induction 125% of all glomeruli presented podocyte adhesions to the Bowmans capsule with otherwise healthy glomeruli (Fig. 1B). At 9 days after induction, 88% of the glomeruli of *Nphs2*^∆pod^ mice were healthy, and in 12% of the glomeruli adhesions to the Bowmans capsule and mesangial cell proliferations were observed. At 17 days after induction only 60% of the glomeruli remained healthy, 16% presented mild damage with adhesions, 13% a moderate damage with additional sclerosis around the Bowmans capsule and 10% with severe damage and additional hyaline deposits^13^. Glomerular injury of mice at 5, 9 and 17 days after FSGS induction, correlated with their urinary protein excretion (Supplementary Fig. 1A). Morphometric analysis of transmission electron microscopy (TEM) images revealed in *Nphs2*^∆pod^ mice in comparison to control at 5 day, 9 days and 17 days after induction that podocytes formed long apical microvilli, pseudocysts and foot process effacement followed by cell detachment (Fig. 1C–G) albeit to different extent. At 17 days after induction additional podocyte flattening was observed (Fig. 1H). Podocyte cell size was determined on confocal images of synaptopodin-stained glomeruli. Podocyte cell hypertrophy was encountered at 9 and 17 days after FSGS induction (Fig. 1I and J).

### Time-dependent assessment of accumulation of lysosomes, lysosomal hydrolases, lysosome transcription response machinery and mTORC1 activity during FSGS development

**Fig. 2:**
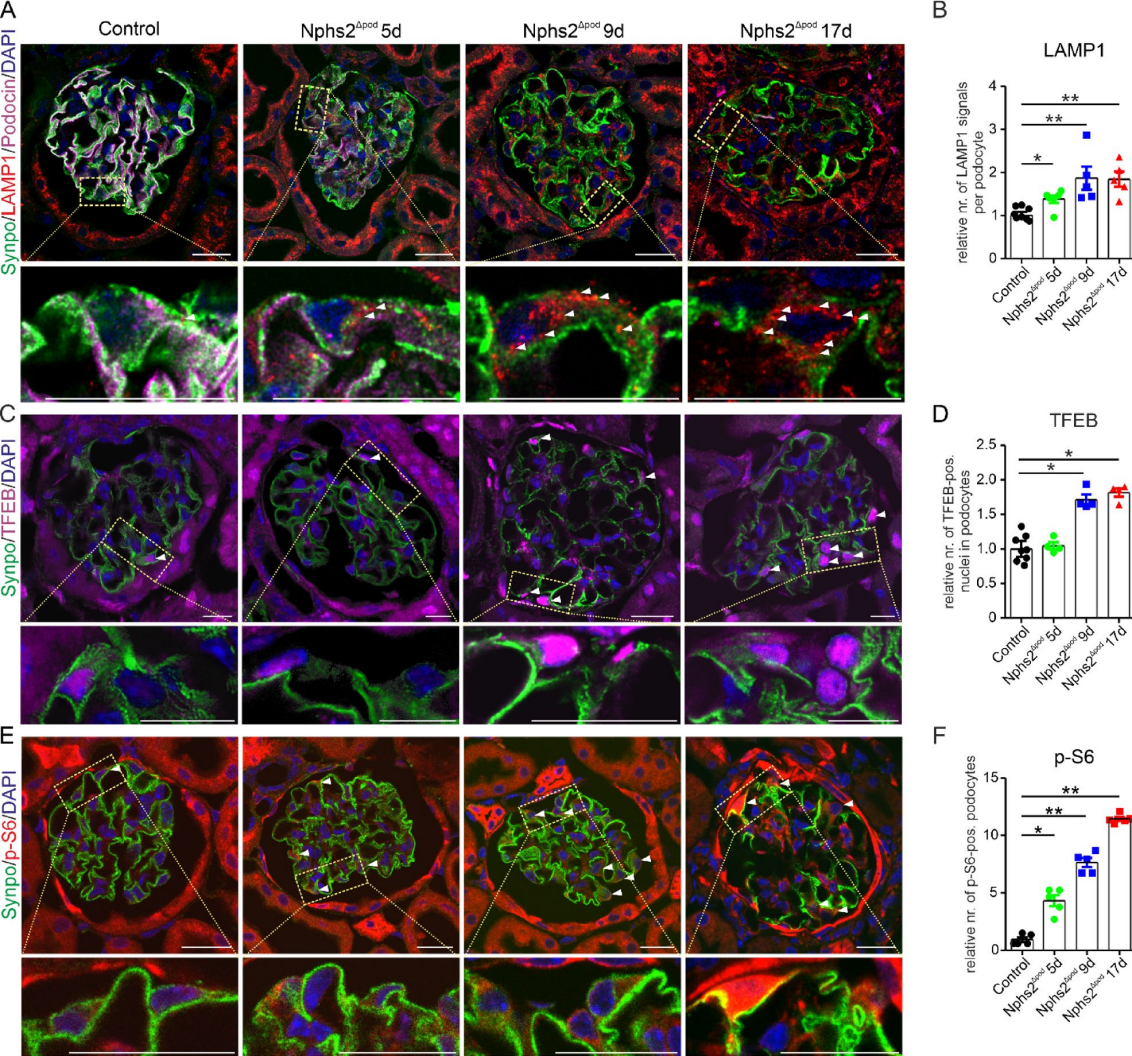
Glomerular damage leads to an early lysosome stress response. (**A** and** B**) Quadruple staining of 5 µm renal cryo sections using anti-synaptopodin (Synpo, green) and anti-podocin (magenta) marking podocytes, anti-LAMP1 (red) stained lysosomes and DAPI (blue) for nuclear staining. Yellow rectangle showing region magnified below. Scale bar = 20 µm. Note the different magnification demonstrated by the scale bar. White arrow heads point to LAMP1-positiv vesicles. For quantification, LAMP1-positive vesicles were determined per podocytes. The mean of the control is set to 1 and compared to *Nphs2*^Δpod^. n = 4–8 per group with > 20 glomeruli per n. *, *P* < 0.05 ; **, *P* < 0.01. (**C** and **D**) Triple staining of 5 µm cryo sections using anti-synaptopodin (Synpo, green) marking podocytes, anti-TFEB (magenta, transcription factor) and DAPI (blue) for nuclear staining. Yellow rectangle showing region magnified below. Scale bar = 20 µm (**C**). Note the different magnification demonstrated by the scale bar. White arrow heads point to TFEB-positive nuclei. Higher magnification images are shown below. For quantification, TFEB-positive nuclei were counted in relation to the number of podocytes. The mean of the control is set to 1 and compared to *Nphs2*^Δpod^ (**D**). n = 4–8 per group with > 20 glomeruli per n. *, *P* < 0.05. (**E** and **F**) Triple staining of 5 µm cryo sections using anti-synaptopodin (Synpo, green) marking podocytes, anti-p-ribosomal protein S6 (p-S6, magenta) and DAPI (blue) for nuclear staining. Yellow rectangle showing region magnified below. Scale bar = 20 µm (**E**). Note the different magnification demonstrated by the scale bar. White arrow heads point to p-S6-positive podocytes. Higher magnification images are shown below. For quantification, p-S6-positive podocytes were counted in relation to the number of podocytes. The mean of the control is set to 1 and compared to Nphs2^Δpod^ (**F**). n = 5–6 per group with > 20 glomeruli per n. *, *P* < 0.05 and **, *P* < 0.01.

Lysosome accumulation is frequently observed in glomerular injuries with foot process effacement^2^. To determine a possible causative involvement of the lysosomes in the disease progression the number of lysosomes per podocyte was determined by immunohistochemical quadruple labeling of lysosomes with anti-LAMP1, podocytes with anti-synaptopodin and anti-podocin and nuclei. Surprisingly, in *Nphs2*^∆pod^ mice already at 5 days and also later on at 9 and 17 days a significant increase in the number of lysosomes was found (Fig. 2A and B). The number of podocyte lysosomes correlated with the podocin expression in an inverse fashion (Supplementary Fig. 2A) and correlated with the urinary protein excretion of *Nphs2*^∆pod^ mice at various time points (Supplementary Fig. 1A). To identify functional lysosomes containing hydrolases, additional quadruple staining was performed for LAMP1, cathepsin B and podocytes and nuclei. Confirming our results, functional lysosomes per podocyte were significantly higher early in FSGS at 5 days after induction and also at later time points (Supplementary Fig. 2C and D). Single channel images are presented in (Supplementary Information 2).

To determine whether lysosomal accumulation is accompanied with an augmentation of the master regulator of lysosomal biogenesis, transcription factor EB (TFEB) and mTORC1 activity, we analyzed the number of TFEB-positive podocyte nuclei (Fig. 2C and D) and phospho-S6-positive podocytes (Fig. 2E and F). Single channel images are presented in (Supplementary Information 2). Compared to control, *Nphs2*^∆pod^ mice displayed a significantly increased TFEB expression starting from day 9 and continued further at day 17 after induction and strong induction of mTORC1 activity already at 5 days after FSGS induction. These results suggests that the observed lysosomal accumulation early in FSGS development is due to an increase in mTORC1 activity and is part of a lysosomal stress response rather than dysregulated lysosomes.

During the analysis of podocyte TEM images, a high number of multivesicular bodies was detected and therefore also assessed at the various time points. The total number of multivesicular bodies (MVB) remained unaltered in Nphs2^∆pod^ mice at 5, 9 and 17 days after FSGS induction compared to control (Supplementary Fig. 1B–D). However, dividing the MVB by their appearance in dark and bright showed in Nphs2^∆pod^ mice at 17 days after induction a significant increase in dark MVB compared to reduced bight MVB.

### Time-dependent assessment of CXCL1, galectin-3, endoplasmic reticulum (ER) and mitochondrial stress during FSGS development

**Fig. 3:**
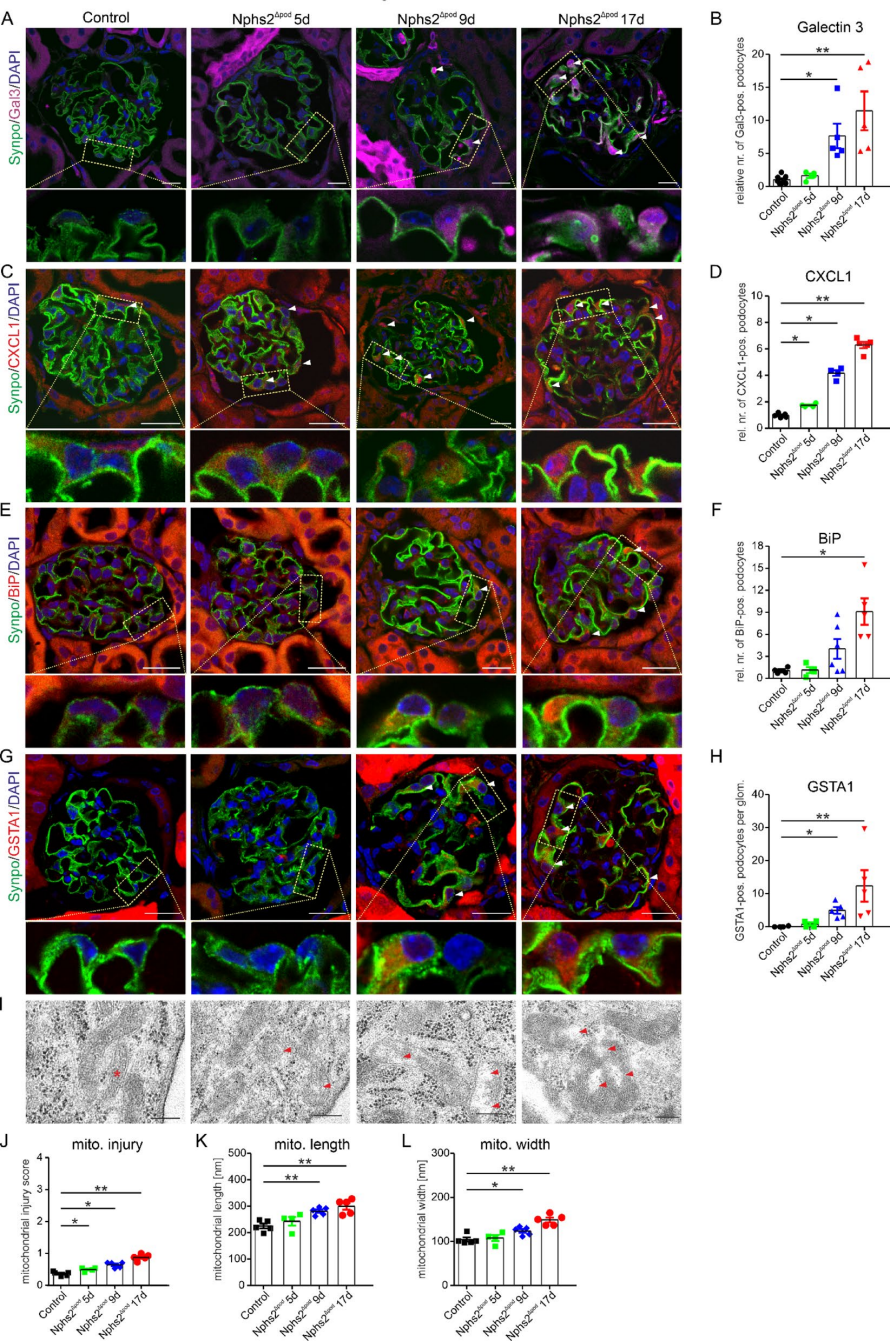
Podocyte damage activates cellular stress response. (**A** and** B**) Triple staining of 5 µm renal cryo sections using anti-synaptopodin (Synpo, green), anti-galectin 3 (Gal3, magenta) and DAPI (blue) for nuclear staining. White arrow heads point to Gal3-positive podocytes. Yellow rectangle showing region magnified below. Scale bar = 20 µm (**A**). For quantification, Gal3-positive podocytes were counted. The mean of the control is set to 1 and compared to *Nphs2*^Δpod^ (**B**). n = 4–6 per group with > 20 glomeruli per n. *, *P* < 0.05 ; **, *P* < 0.01. (**C** and **D**) Triple staining of 5 µm renal cryo sections using anti-synaptopodin (Synpo, green), anti-CXCL1 (magenta) and DAPI (blue) for nuclear staining. White arrow heads point to CXCL1-positive podocytes. Yellow rectangle showing region magnified below. Scale bar = 20 µm (**C**). Note the different magnification demonstrated by the scale bar. For quantification, CXCL1-positive podocytes were counted. The mean of the control is set to 1 and compared to Nphs2^Δpod^ (**D**). n = 4–6 per group with > 20 glomeruli per n. *, *P* < 0.05 ; **, *P* < 0.01. (**E** and **F**) Triple staining of 5 µm renal cryo sections using anti-synaptopodin (Synpo, green), anti-BiP (red) and DAPI (blue) for nuclear staining. White arrow heads point to BiP-positive podocytes. Yellow rectangle showing region magnified below. Scale bar = 20 µm (**E**). Note the different magnification demonstrated by the scale bar. For quantification, BiP-positive podocytes per glomerulus were counted. The mean of the control is set to 1 and compared to *Nphs2*^Δpod^ (**F**). n = 4–6 per group with > 20 glomeruli per n. *, *P* < 0.05. (**G** and **H**) Triple staining of 5 µm renal cryo sections using anti-synaptopodin (Synpo, green), anti-GSTA1 (red) and DAPI (blue) for nuclear staining. White arrow heads point to GSTA1-positive podocytes. Yellow rectangle showing region magnified below. Scale bar = 20 µm (**G**). Note the different magnification demonstrated by the scale bar. For quantification, GSTA1-positive podocytes per glomerulus were counted. The means were depicted in the graph (**H**). n = 4–5 per group with > 20 glomeruli per n. *, *P* < 0.05 ; **, *P* < 0.01. (**I**) Representative electron microscopy images of podocyte mitochondria obtained from control and Nphs2^Δpod^ groups. Red asterisk labels healthy mitochondria, red arrow heads label vacuole formation. Scale bar = 200 nm. (**J**–**L**) Quantification of mitochondria injury score (**J**), mitochondria (mito) width (**K**) and mitochondria (mito) length (**L**). n = 5 per group with > 22 podocytes evaluated. *, *P* < 0.05 ; **, *P* < 0.01.

To determine whether lysosomal damage and consecutive lysosomal membrane permeabilization may occur, we stained kidney section with anti-galectin-3, a protein known to traffic to the inner lysosomal lumen^14^ facilitating their autophagic degradation by lysophagy. In controls and at 5 days *Nphs2*^∆pod^, no positive signals were observed for galectin-3. In *Nphs2*^∆pod^ mice at 9 days and more pronounced at 17 days after induction, strong cytoplasmic galectin-3 signals were found in the whole podocyte and was significantly increased over the time of FSGS development (Fig. 3A and B) and single channel images are presented in (Supplementary Information 3). The cytoplasmic expression pattern for galectin-3 and the correlation with the degree of podocyte injury suggest a role in inflamed podocytes rather than lysophagy (Supplementary Fig. 3A). In addition, galectin-3 was only rarely co-localized with lysosomes (Supplementary Fig. 3B). CXCL1 is a pro-inflammatory chemokine which is a NF-κB-dependent potent neutrophil chemoattractant and was shown to be highly produced by renal podocytes determined from biopsies from patients with FSGS^15^. In comparison to control, in *Nphs2*^∆pod^ mice CXCL1 expression started to increase significantly at 5 days after induction and further increased at 9 days and at 17 days after induction (Fig. 3C and D). Single channel images are presented in (Supplementary Information 3). This finding was confirmed by in situ hybridization of *cxcl1* mRNA (Supplementary Fig. 3C and D). ER and mitochondria stress were associated with FSGS development and presented to crosstalk with lysosomes^16–19^. Therefore, we determined the time-dependent development of podocyte ER and mitochondria stress using immunoglobulin heavy chain binding protein (BiP also known as GRP78) as sensor for ER stress, glutathione S-transferase alpha 1 (GSTA1) as marker for mitochondrial stress and morphological assessment of mitochondria. In *Nphs2*^∆pod^ mice, BiP starts to be higher expressed in podocytes after 9 days of FSGS induction and was significantly augmented at 17 days of FSGS (Fig. 3E and F). Single channel images are shown in (Supplementary Information 3). In comparison to control, *Nphs2*^∆pod^ mice displayed a significant increase in GSTA1 at 9 day and even more pronounced at 17 days after induction (Fig. 3G and H, and Supplementary Information 3). We further analyzed the mitochondria injury and mitochondria length and width using TEM images of podocytes obtained from FSGS mouse model at 5, 9 and 17 days (Fig. 3I–L). Mitochondria injury started at 5 days after FSGS induction and further increased at 9 and 17 days. At the beginning of mitochondria injury, vacuole formation was evident and membrane fissures later at 17 days after FSGS induction. In comparison to mitochondria injury, mitochondrial length and width changed later at 9 days and more pronounced at 17 days after FSGS induction.

### Cell stress induced lysosomal stress response

**Fig. 4:**
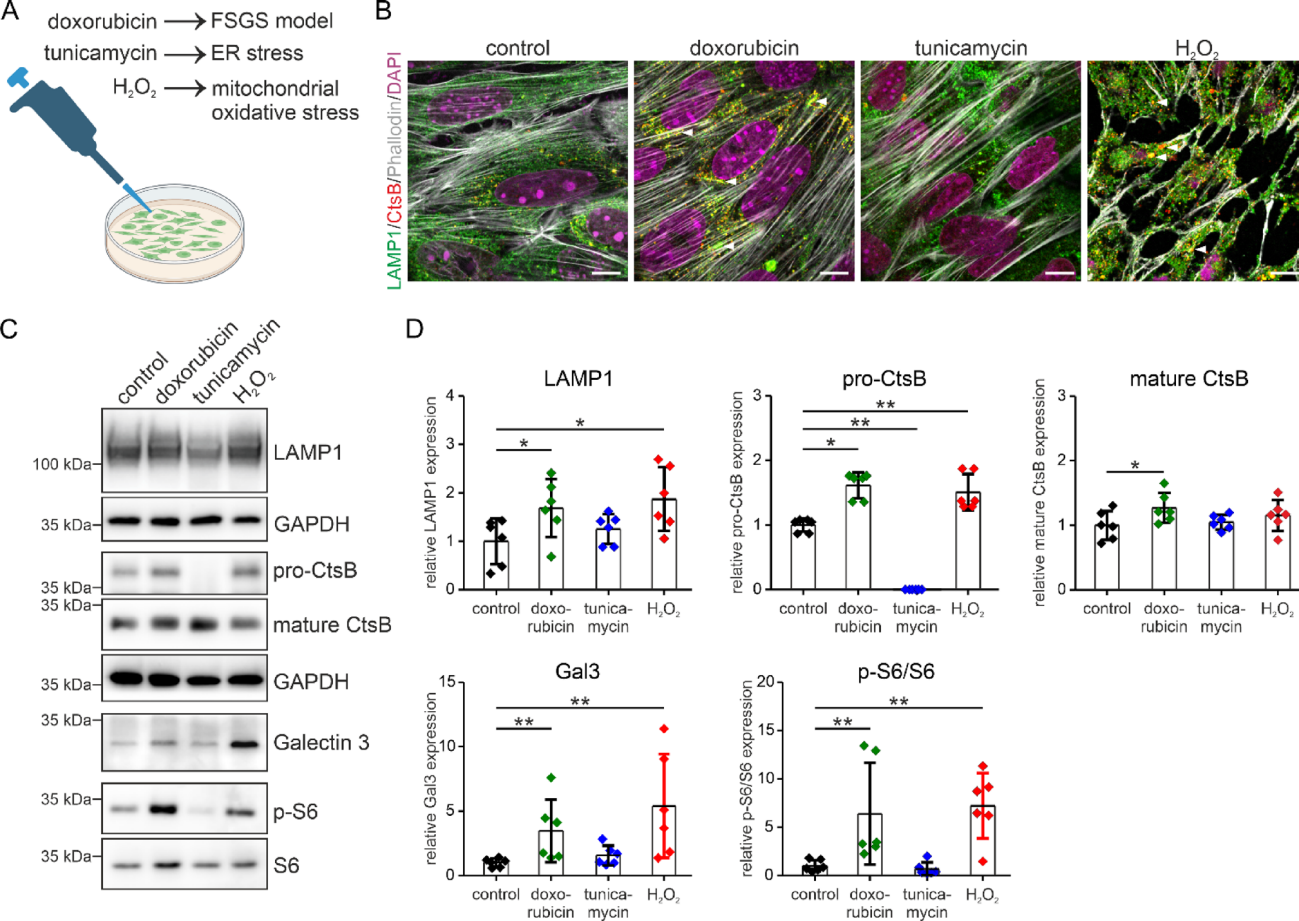
Cell stress induces a lysosome stress response***.*** (**A**) Visualisation of experimental setting with immortalized podocytes. (**B**) Quadruple staining of immortalized podocytes using anti-LAMP1 (green) to mark lysosomes, anti-cathepsin B (CtsB, red), Phalloidin (grey) to illustrate actin filaments and DAPI (blue) for nuclear staining. Scale bar = 10 µm. White arrow heads point to the accumulation of lysosomes frequently observed along stress fibers. (**C** and **D**) Western blot analyses of LAMP1, cathepsin B (CtsB), galectin-3 (Gal3), p-ribosomal S6 protein (p-S6) and ribosomal S6 protein (S6) expression in control and 0.5 µg/ml doxorubicin-, 0.5 µg/ml tunicamycin- and 50 µM H_2_O_2_-treated podocytes (**C**). GAPDH served as loading control. Densitometrical quantification of respective western blots are shown (**D**). The mean of the control is set to 1. n = 6. Original blots are available as Supplementary Information 1. *, *P* < 0.05 ; **, *P* < 0.01.

During FSGS development we observed various cell stresses appearing in podocytes. To answer the question which cell stress may induce or influence lysosomal stress response in podocytes, differentiated podocytes were stimulated with doxorubicin for FSGS induction, with H_2_O_2_ for mitochondrial stress and with tunicamycin for ER stress (Fig. 4A). In comparison to control and tunicamycin-induced ER stress, doxorubicin and H_2_O_2_ induced a lysosomal stress response by increasing the number of lysosomes and the abundance of LAMP1 and of the lysosomal hydrolase cathepsin B (Fig. 4B–D). In addition, galectin-3 and mTORC1 activity, as determined by the phosphorylation of protein S6, were significantly higher upon doxorubicin and H_2_O_2_ treatment (Fig. 4C and D). Successful ER stress induction is presented in Supplementary Fig. 4.

## Discussion

Our study revealed that mTORC-1 induced lysosome stress response and mitochondrial injury are the earliest events during FSGS development. Podocytes are strongly dependent on lysosome function because of the important intracellular turnover necessary for cell survival and longevity of postmitotic cells. An upregulation of lysosomal proteins and transcripts were observed in glomeruli of patients with FSGS^20^^,^^21^. The resulting alteration in protein homeostasis (proteostasis) may be considered as a conserved key mechanism in FSGS and other proteinuric kidney diseases^7^. Lysosomes are key organelles for nutrient sensing recruiting mTORC1 to the outer membrane upon lysosomal amino acid release. Lysosome accumulation correlated negatively with the decline in podocin expression. Loss of podocin was presented leading to mechanical stress acting on podocytes^22^. To cope with mechanical stress, podocytes need to replace intracellular filaments and contact proteins with higher protein turnover which may release amino acids for mTORC1 activation^23^^,^^24^. Activated mTORC1 was shown to phosphorylate lysosomal mTORC1 substrates, such as TFEB^4^. TFEB, known as important regulator of lysosomal biogenesis and autophagy, also regulates podocyte actin cytoskeleton^25^. Therefore, lysosomal activation may cope with mechanical stress acting on podocyte cytoskeleton^24^ during FSGS development.

Despite the importance in protein degradation and nutrient sensing, lysosomes are key organelles for innate and adaptive immunity^26^. For the innate immune response, lysosomal membranes express the toll-like receptors (TRL)7, TRL8 and TRL9^27^. TRL7 and TRL8 respond to single-stranded RNA and TRL9 can be activated by DNA with unmethylated CpG dinucleotides. Activation of TLR7-9 are known to result into a conformational change with subsequent activation of nuclear factor-kB (NF-kB) and transcription of proinflammatory cytokines and chemokines^27^. In our study, we have observed CXCL1 protein and *cxcl1* mRNA generation which were increased to a similar extent as the number of lysosomes already at 5 days after FSGS induction. Interestingly, chemokines CXCL1 – 3, -5 and -8 were shown to be specifically processed by cathepsins with resulting higher chemotactic activity of the truncated forms^28^. Furthermore, cathepsin B is also able to activate NOD-, LRR-, and pyrin domain-containing protein 3 (NLPR3) inflammasome with consecutive pyroptosis^29^ underlining the role of lysosomes and their cathepsins in inflammatory response during FSGS development. Doxorubicin is frequently used to initiate FSGS in animal and cell culture models including in our cell culture experiments^30^. Remarkably, it was shown to display both nuclear and lysosomal distribution after cell uptake causing massive DNA damage, in response pro-inflammatory signaling mediated by NF-κB and STAT3 and consequent CXCL1 secretion^31^. Galectin-3 is a member of beta-galactoside-binding animal lectins, and induces the secretion of proteases such as lysosomal cathepsin B^32^. It was further shown that galectin-3 is able to bind infiltrated neutrophils and to promote NLPR3 inflammasome formation^33^. Therefore, galectin-3 expression at 9 days after FSGS induction may contribute to the observed inflammation.

Analog to mTORC-1-induced lysosomal stress response, mitochondria injury occurred at 5 days after FSGS induction. Most frequently, loss of the inner mitochondria membrane, also called vacuole formation, was observed at 5 days. Inner mitochondria membrane disruption was presented to be due to calcium overload, oxidative stress or ATP depletion^34–36^. Altered intracellular calcium handling may also be due to mechanical stress-induced calcium signaling^37^. Mitochondria stress contributes to the observed lysosome stress response and was shown to initiate inflammasome response^38^. In our study we found that mitochondrial dysfunction occurred at the onset of CXCL1 expression. Mitochondria dysfunction may therefore be part of disease initiation and mitochondria-targeted therapy have been found beneficial in FSGS treatment^17^. Podocytes possess also a highly efficient endoplasmic reticulum (ER) protein-folding capacities rendering them sensitive to endoplasmic reticulum stress, which was shown to play a role in podocyte injury in FSGS^39^. From our results however, ER stress does not assist to the early lysosomal stress response rather it may conserve FSGS injury. Although we cannot transfer our in vitro data to the in vivo situation, limiting our study.

During TEM image analysis we have observed the existence of many MVB containing extracellular vesicles (EV´s). In inflammatory diseases, EV´s may contain NLPR3 and their release may contribute to the inflammatory spreading occurring in FSGS^40^. In our study, we did not detect alterations in total number of MVB during the time. However, at 17 days after FSGS induction a division of MVB in dark versus bright MVB revealed a significant higher number of dark MVB compared to bright MVB. This altered MVB appearance may result from altered cargo content and needs further exploration in future.

In conclusions, lysosomal stress response and mitochondria dysfunction are the earliest events in FSGS development. Both organelles having a role in signaling and inflammation, may facilitate the initiating event of inflammation within the podocyte. The lysosome and mitochondria stress coincides with podocytes undergoing foot process effacement appearing with early signs of their activated inflammatory state, the apical microvilli transformation. From our result, we would propose the exploration of circumventing lysosome and mitochondria dysfunction to prevent progression of FSGS.

## Material and methods

### Animals, fixation and tissue processing for immunohistochemistry

All animal experiments were conducted and reported according to the NIH Guide for the care and use of Laboratory animals, as well as the Swiss and German law for the welfare of animals and were approved by local authorities (Cantonal Veterinary Office, Canton of Fribourg, Switzerland: 2013_06E_FR, 23614; 2016_28_FR, 28328; Ministry of Energy, Agriculture, the Environment, Nature and Digitalization of Schleswig–Holstein, Germany: V242-20597/2018). All animal experiments were carried out in accordance with the ARRIVE guidelines. Mice were housed in a SPF facility with free access to chow and tap water and a 12 h day/night cycle. Mice were initially obtained from the Mouse Clinical Institute (Institut Clinique de la Souris, Illkirch, France)^12^. Breeding and genotyping was performed as described^12^^,^^13^. *Nphs2*^fl/fl^ (control) and *Nphs2*^fl/fl^ crossbred with inducible podocyte-specific Cre recombinase transgenic mice, termed *Nphs2*^∆pod^, were used^12^^,^^13^. Mice of mixed gender in an SV129 PAS background were used. For the induction of knockout leading to focal segmental glomerulosclerosis, 6 weeks old *Nphs2*^∆pod^ and control mice received tamoxifen (33 mg/kg per d for 5 d; Sigma, Buchs, Schweiz) by daily *i.p.* injection for 4 days in the evening after. Mice were anesthetized using a combination of *i.p.* injected ketamine (120 mg /kg) and xylazine (16 mg/kg) and kidneys were perfused retrogradely using 4% PFA in PBS. Perfusion-fixed specimens were postprocessed for cryo-, paraffin-, and epon-embedding for further histochemical, light and electron microscopy analysis.

### Cell culture experiments

Immortalized podocytes (SVI; CLS Cell Line Service GmbH, Eppelheim, Germany) were handled as described in^41^. Briefly, podocytes were maintained in RPMI-1640 medium (Sigma-Aldrich, St. Louis, MO, USA) supplemented with 10% fetal bovine serum (FBS), 100 U/ml penicillin, and 0.1 mg/ml streptomycin (Thermo Fisher Scientific, Waltham, MA, USA). To propagate podocytes, we cultivated cells at 33 °C. To induce podocyte differentiation, we maintained podocytes at 37 °C for at least two weeks before applying doxorubicin (15007, Cayman, final concentration 0.5 µg/ml), tunicamycin (T7765, Sigma-Aldrich, 0.5 µg/ml) or H_2_O_2_ (8070.1, Roth, Karlsruhe, Germany, 50 µM) for 24 h.

### Immunocyto- and immunohistochemistry

Renal cryo- or paraffin sections of 5 µm thickness were permeabilized using 0.5% tritonX-100/PBS for 30 min, blocked with 5% skim milk/PBS for 1 h and incubated with primary antibody overnight at 4 °C. After washing, cells were incubated with suitable secondary antibodies for 2 h at room temperature. Nuclei were stained using 4’,6-diamidino-2-phenylindole (DAPI, Cat # D1306, Thermo Fisher). Images were acquired using Facility Line (Abberior Instruments, Göttingen, Germany) with Olympus IX83 microscope (Hamburg, Germany) and Imspector software (Abberior Instruments, Göttingen, Germany). For quantification, images were merged and positive signals were evaluated by an independent observer.

To determine podocyte cell size, synaptopodin-stained sections were evaluated. Each podocyte cell body per glomeruli was surrounded and the area was quantified using Fiji Image J. The mean podocyte cell size per mouse represents the mean of all glomeruli analyzed per mouse.

### Morphometric assessment of glomerular injury

A scoring system was applied to periodic acid-Schiff- (PAS-) stained 5 µm paraffin sections to evaluate the extent of glomerular injury as described in^42^^,^^43^. Signs of adhesions, thickening of the glomerular basement membrane, glomerular sclerosis, podocyte hypertrophy, mesangial expansion, collapsed capillaries, and hyaline deposits were assessed. For each glomerulus the semi-quantitative score was applied with 0 = no lesion, 1 = up to 25% of the glomerulus was affected, 2 = up to 50%, 3 = up to 75% and 4 = up to 100%. An average score for the whole kidney were calculated. The analyses were performed in a blinded fashion. Ultra-thin sections of epon-embedded kidney tissue were stained using uranyl acetate and lead citrate. Transmission electron microscopy (TEM; JEM 1400 plus, JOEL, and TemCam F416, TVIPS) were used to analyze podocyte’s morphology. In total, n > 22 podocytes were analyzed per animal. Podocytes demonstrating the formation of microvilli, pseudocysts, cell detachment and flattening were quantified as number of positive podocytes in percent of all podocytes analyzed, as described^2^. Foot process effacement was determined by applying a scoring method to evaluate the degree of foot effacement around the capillaries with score 0: no effacement, 1 = up to 25%, 2 = up to 50%, 3 = up to 75% and 4 = up to 100%.

Mitochondria analysis. Mitochondria length and width were determined using TEM pictures of 20 different podocytes per mice. Mitochondria injury score was quantified according to the method established by Kayhan et al.^44^. Changes in the number and morphology of cristae, formation of vacuoles, presence of cracks/fissures and presence of myelin figures were assessed. The scoring system was established with 0: no injury, 1 = up to 25%, 2 = up to 50%, 3 = up to 75% and 4 = up to 100%. Approximately around 200—300 podocyte mitochondria were analyzed per mouse.

### RNA in situ hybridization

Die RNA in situ hybridization against *cxcl1* mRNA was performed using RNAscope™ (RNAscope™ 2.5 HD Reagent Kit, Advanced Cell Diagnostics Bio-Techne, Wiesbaden-Nordenstadt, Germany) and probe against mouse *cxcl1* (RNAscope Probe Mm-Cxcl1, 407721, Advanced Cell Diagnostics Bio-Techne). RNAscope™ was carried out according to manufacturer’s instructions. To verify mRNA quality, a probe against the housekeeping gene peptidylprolyl isomerase B (cat. no.313911, accession no. NM_011149.2) served as positive control. As negative control served a probe against the bacterial gene DapB (cat.no. 312039, accession no. EF191515). Sections were counterstained with DAPI (4′,6-Diamidin-2-phenylindol, Cat # D1306, Thermo Fisher). Images were acquired using Keyence BZx800 microscope (Keyence, Neu-Isenburg, Germany). The number of *cxcl1*-positive signals were evaluated by an independent observer using merged images.

### SDS-PAGE and immunoblotting

Proteins were isolated using RIPA buffer containing protease inhibitor cocktail (Roche, Basel, Switzerland), separated using SDS gel electrophoresis on polyacrylamide gels and transferred onto nitrocellulose membranes. Protein loading were verified by staining the membrane with 0.1% Ponceau red. Membranes blocked with 5% skim milk/TBS for 1 h and incubated with primary antibodies overnight at 4 °C. After incubation with HRP-conjugated secondary antibodies (Dianova, Hamburg, Germany), immunoreactive bands were detected by chemiluminescence using Immobilon Western HRP substrate (Millipore, Darmstadt, Germany) and the chemiluminescence imaging system Azure 300 (Biozym Scientific, Hessisch Oldendorf, Germany). Signal density were analyzed using ImageJ software. Of note, membranes were cut prior blocking and hybridization with antibodies.

### Antibodies

The following antibodies were used: guinea pig anti-synaptopodin (PROGEN Biotechnik GmbH, Heidelberg, Germany), rat anti-LAMP1 (ID4B-c, Developmental Studies Hybridoma Bank, Iowa City, USA), goat-anti mouse Cathepsin B (AF965, R&D, Systems, Minneapolis, USA), rabbit anti-TFEB (Cell Signaling Technology, Frankfurt am Main, Germany), goat anti-Galectin 3 (R&D Systems), rabbit anti-GSTA1 (14475–1-AP, Proteintech, Planegg-Martinsried, Germany), rabbit anti-BiP (3177, Cell Signaling Technology), rabbit anti-GAPDH (10494–1-AP, Proteintech), rabbit anti-protein S6 (2217, Cell Signaling Technology), rabbit anti-phospho-S6 (5364, Cell Signaling Technology), rabbit anti-podocin (P0372, Sigma Aldrich), anti-CXCl1 (12335–1-AP, Proteintech).

Secondary antibodies used were coupled with HPR, Alexa647, Alexa594 or Alexa488 were purchased from Dianova (Hamburg, Germany), coupled with StarRED or Star580 either from Abberior GmbH (Göttingen, Germany) or donkey-derived secondary antibodies (Dianova) were coupled with Star580 or StarRed as described^45^. Actin filaments were stained with Acti-Stain 488 phalloidin (PHDG1-A, Cytoskeleton, Denver, USA).

### Statistics

Statistical comparisons were performed with the GraphPad Prism Software Package 5 (GraphPad Software, La Jolla, CA, USA) using the Kruskal–Wallis test following Dunns post test *p* values of < 0.05 were judged statistically significant. Asterisks are used in the figures to explicitly demonstrate the statistical significance (*, *P* < 0.05; **, *P* < 0.01; ***, *P* < 0.001).

## Supplementary Information

Below is the link to the electronic supplementary material.

Supplementary Material 1

Supplementary Material 2

## Data Availability

The data that support the findings of this study are available in the Materials and Methods, Results, and/or Supplemental Material of this article.
