# Supplementary material for: Lysosome stress response and mitochondria injury are the earliest detectable alteration in FSGS
Source: Sci Rep. 2025 Oct 13;15:35570. doi: 10.1038/s41598-025-22622-x (PMC12518675; doi:10.1038/s41598-025-22622-x)
Supplement: Supplementary file 1 — Supplementary Material 1 [file 41598_2025_22622_MOESM1_ESM.docx]

***
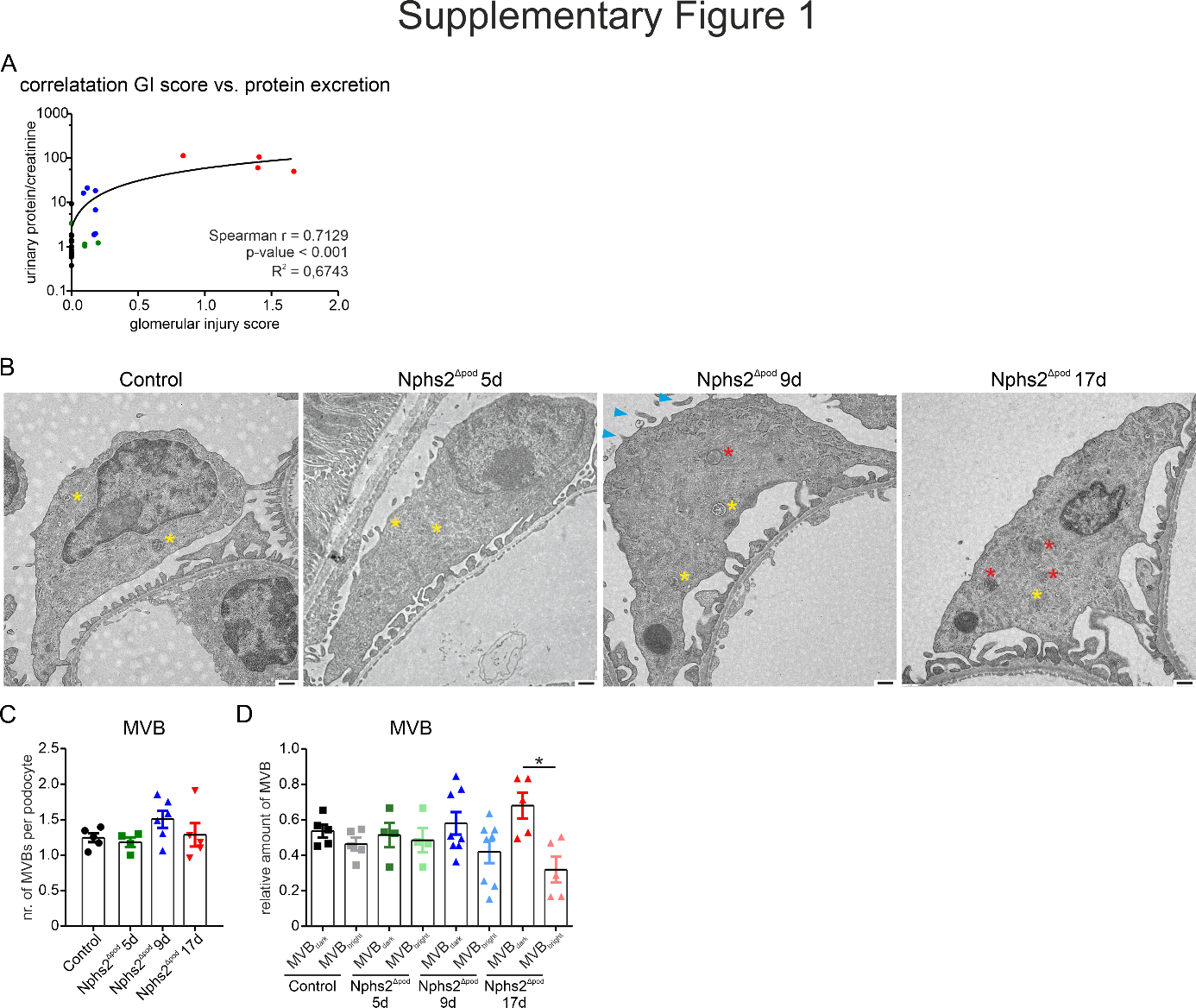
***

***Supplementary Fig. 1: Correleation of glomerular injury with proteinuria and cell stress altering the abundance of MVB appearance in podocytes.*** (**A**) Correlation of glomerular injury score with urinary protein excretion of control (black circle), 5 days FSGS (green circle), 9 days FSGS (blue circle) and 17 days FSGS (red circle). (**B**) Electronmicroscopy images of podocytes from control and *Nphs2*^Δpod^ mice. Red asterisk mark dark multivesicular bodies (MVB), yellow asterisk bright MVB, blue arrow heads point to microvilli. Scale bar = 500 nm. (**C**) Quantification of MVB abundance in podocytes. The number of MVB per podocyte was assesses. n = 4 – 8 per group. *, *P* < 0.05. (**D**) Quantification of the abundance of bright and dark MVB in podocytes. The mean total number of MVB was set to 1 and the relative distribution is depicted in the graph. n = 5 – 8 per group. *, *P* < 0.05.

***
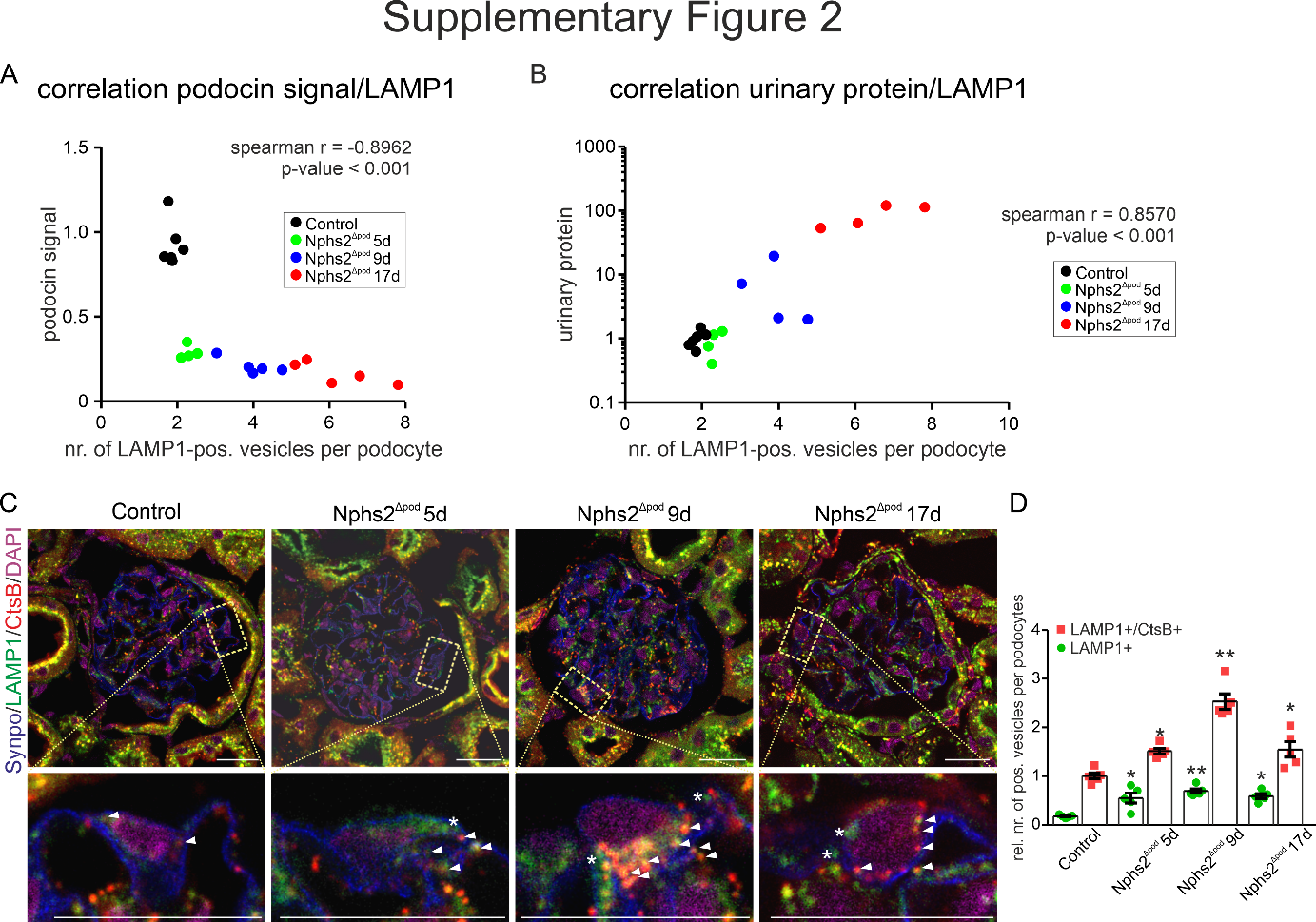
***

***Supplementary Fig. 2:*** ***Correlation of LAMP1 with podocin, of LAMP1 with urinary protein and colocalization of LAMP1 and cathepsin B*.** (**A**) Correlation of podocin expression (in relation to synaptopodin signal representing the amount of all podocytes) with the number of LAMP1-positive vesicles in the podocytes of control (black circle), 5 days FSGS (green circle), 9 days FSGS (blue circle) and 17 days FSGS (red circle). (**B**) Correlation of urinary protein excretion (in relation to urinary creatinine) with the number of LAMP1-positive vesicles in the podocytes of control (black circle), 5 days FSGS (green circle), 9 days FSGS (blue circle) and 17 days FSGS (red circle). (**C** and **D**) Quadruple staining of 5 µm renal cryo sections using anti-synaptopodin (Synpo, blue), anti-cathepsin B (CtsB, red), anti-LAMP1 (green) staining lysosomes and DAPI (blue) for nuclear staining. Yellow rectangle showing region magnified below. Scale bar = 20 µm. Note the slightly different magnification demonstrated by the scale bar. White arrow heads point to LAMP1+/CtsB+ double positive vesicles, white asterisk mark LAMP1-single positive vesicles. The mean of LAMP1+/CtsB+ double positive vesicles of the control is set to 1 and compared to *Nphs2*^Δpod^. The mean of LAMP1+ single positive vesicles are in relation to LAMP1+/CtsB+ double positive vesicles and compared to the respective *Nphs2*^Δpod^. n = 5 per group with >20 glomeruli per n. *, *P* < 0.05 ; **, *P* < 0.01.

***
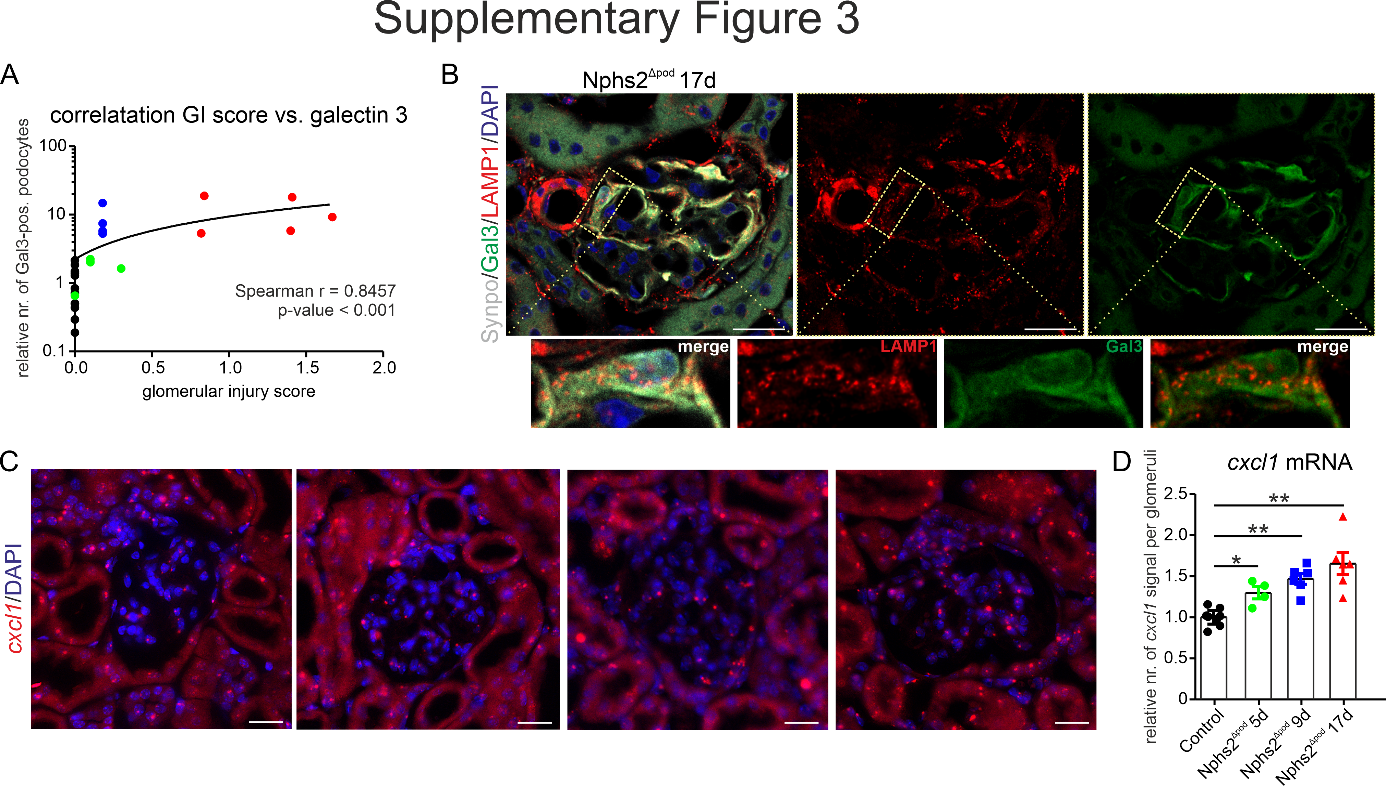
***

***Supplementary Fig. 3:*** ***Single channel images to Figure 3, correlation of galectin-3 with glomerular injury and RNAscope of glomerular cxcl1 mRNA******.*** (**A**) Correlation of glomerular injury score with podocyte galectin-3 expression at 5 (green circle), 9 (blue circle), and 17 days (red circle) after FSGS induction compared to control (black circle). (**B**) Representative image of quadruple staining of 5 µm renal cryo sections using anti-synaptopodin (Synpo, grey), anti-galectin 3 (Gal3, green), anti-LAMP1 (red) staining lysosomes and DAPI (blue) for nuclear staining from *Nphs2*^Δpod^ 17d showing nearly absent of galectin 3/LAMP1 colocalization. Yellow rectangle showing region magnified below. Scale bar = 20 µm. (**C** and **D**). For quantification, *cxcl1 mRNA*-corresponding red signals within the glomeruli were counted. The mean of the control is set to 1 and compared to Nphs2^Δpod^ (**D**). n = 4 – 6 per group with > 50 glomeruli per n. *, *P* < 0.05 ; **, *P* < 0.01.

**
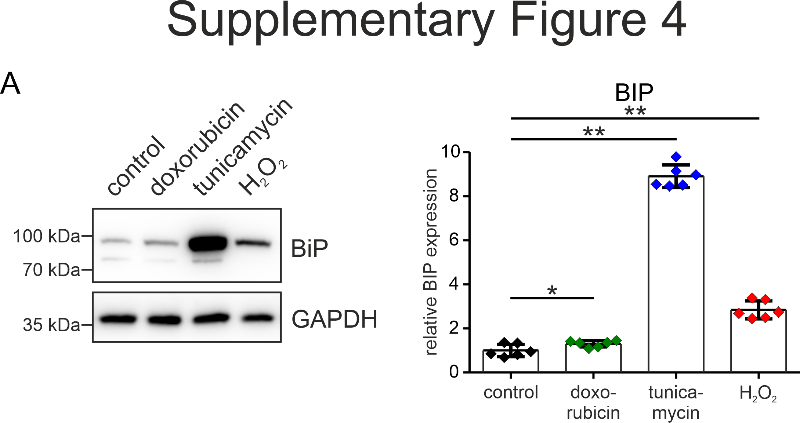
**

**Supplementary Fig. 4: *ER-stress induction in podocytes.*** Western blot analysis of BiP in control and 0.5 µg/ml doxorubicin-, 0.5 µg/ml tunicamycin- and 50 µM H_2_O_2_-treated podocytes (*left*). GAPDH served as loading control. Densitometrical quantification is shown in the graphs (*right*). The mean of the control is set to 1. n = 6. Original blots are available as **Supplementary information**. *, *P* < 0.05 ; **, *P* < 0.01.
