# Supplementary material for: Lysosome stress response and mitochondria injury are the earliest detectable alteration in FSGS
Source: Sci Rep. 2025 Oct 13;15:35570. doi: 10.1038/s41598-025-22622-x (PMC12518675; doi:10.1038/s41598-025-22622-x)
Supplement: Supplementary file 2 — Supplementary Material 2 [file 41598_2025_22622_MOESM2_ESM.docx]

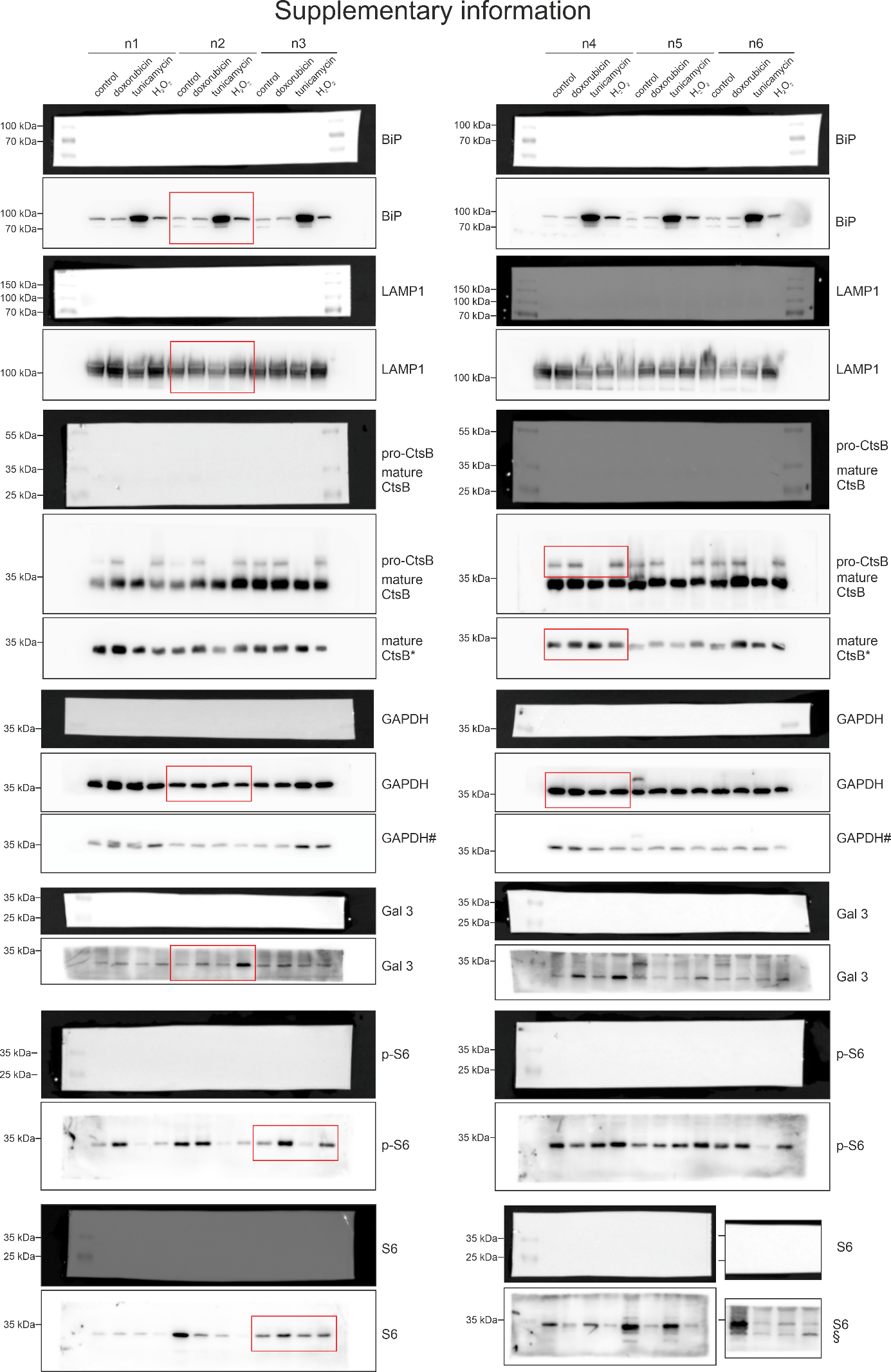


***Supplementary Information 1: Original western blot analysis of each sample.*** The blots were cut into pieces prior hybridization to detect different proteins on the same membrane. Uncropped, unmodified blots are shown. Red rectangles surround regions used for representative images in Figure 4 and Supplementary Figure 2. * for evaluation of mature CtsB exposure time of the same blots were reduced; # lower exposure time of GAPDH blots; § galectin-3 blot was reincubated with S6 antibody (upper band) after detection of galectin-3 (lower band §).


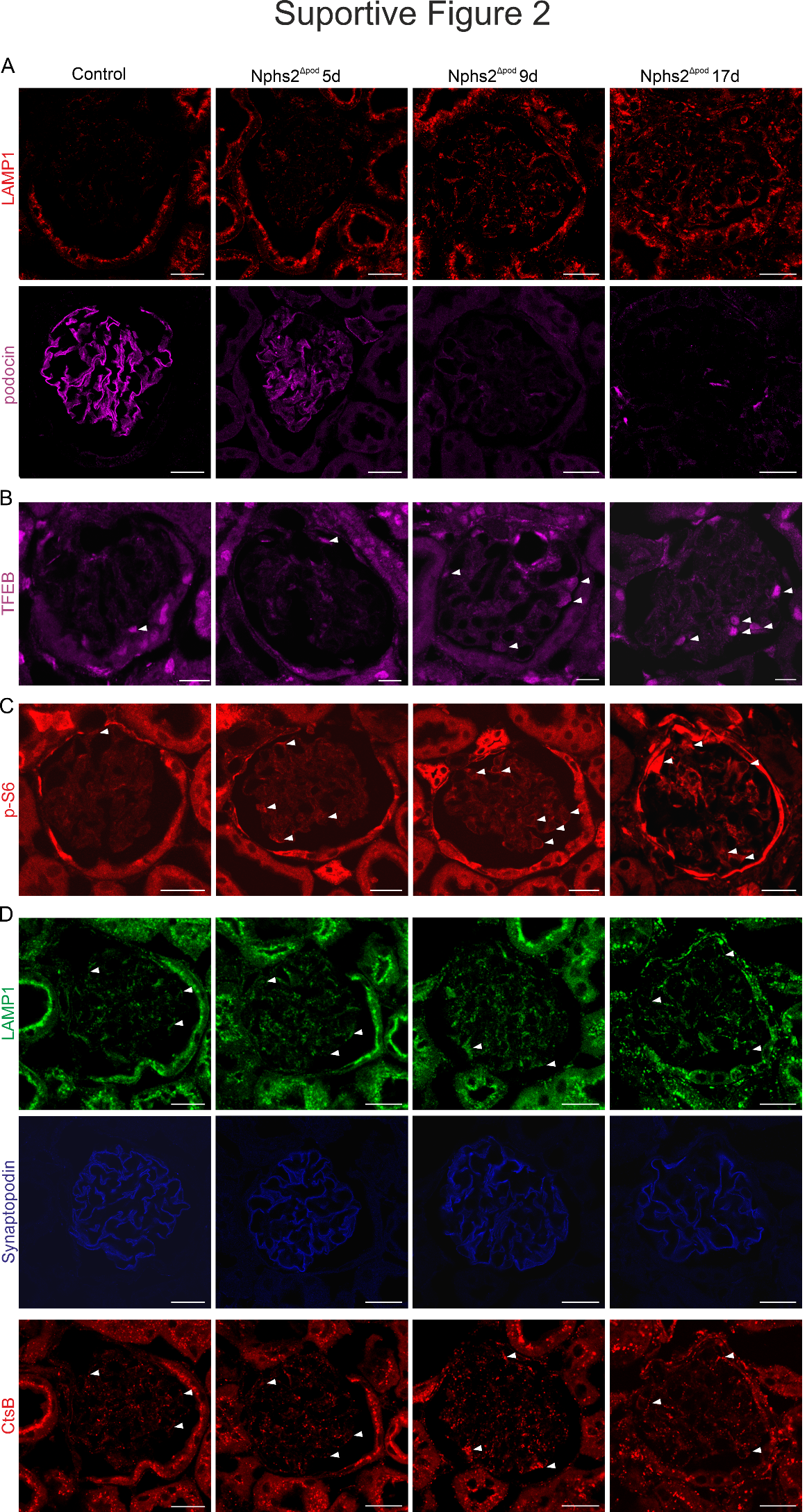


***Supplementary Information 2: Single channels of immunostainings of Fig. 2 and Supplementary Fig. 2.*** Single channels of quadruple/triple staining of **Fig. 2** with (**A**) anti-LAMP1 (red), anti-podocin (magenta), (**B**) anti-transcription factor EB (TFEB, magenta), (**C**) anti-pS6 (red). Single channels of quadruple staining of **Supplementary** **Fig. 2** with (**D**) anti-LAMP1 (green), anti-synaptopodin (Synpo, blue) and cathepsin B (CtsB, red). Scale bar = 20 µm. Note the different magnification demonstrated by the scale bar. White arrow heads point to podocytes positive for the stained protein, respectively.


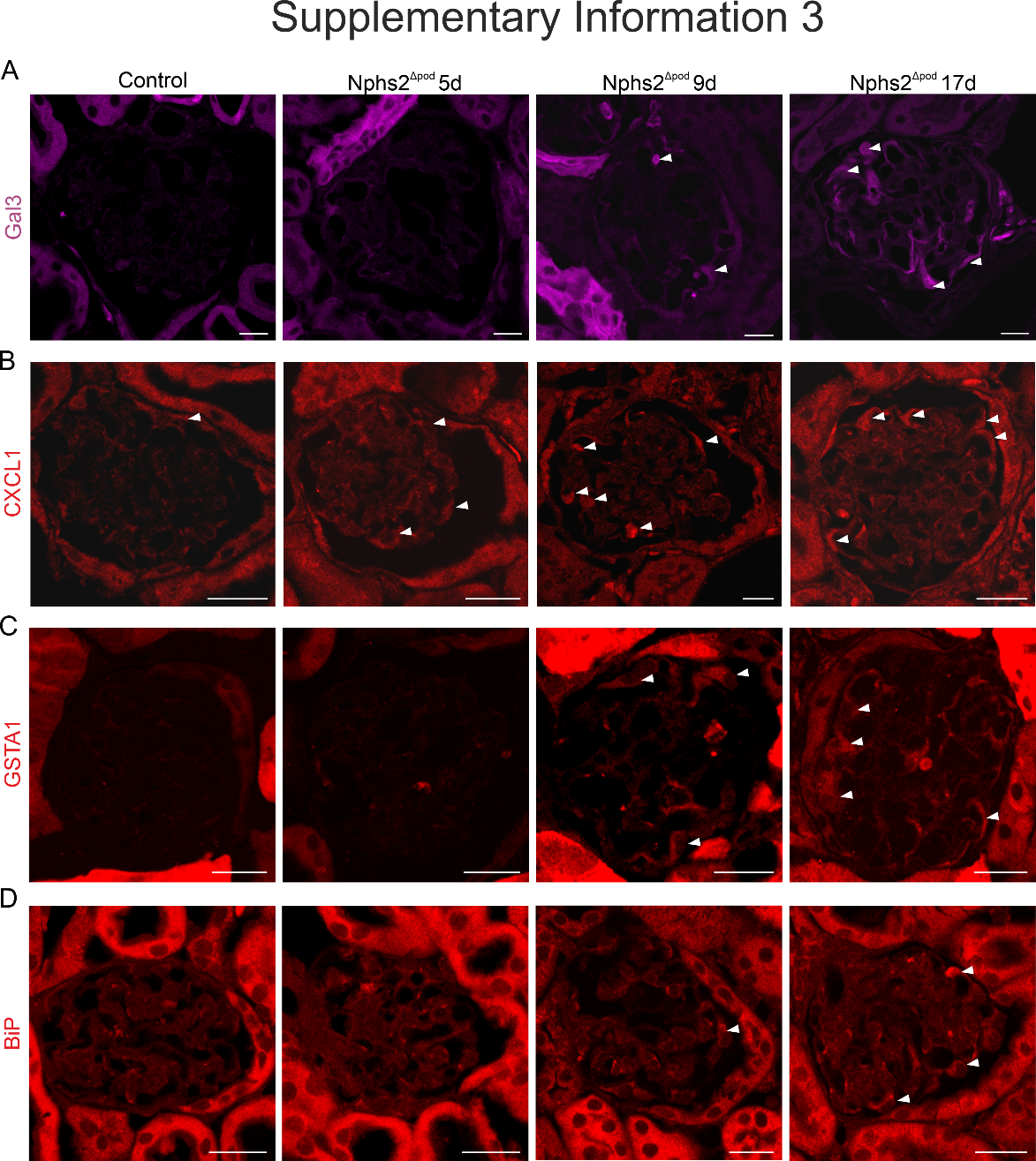


***Supplementary Information 3: Single channels of immunostainings of Fig. 3.*** Single channels of triple staining of **Fig. 3** with (**A**) anti-galectin 3 (Gal3, magenta), (**B**) anti-CXCL1 (red), (**C**) anti-GSTA1 (red) and (**D**) anti-BiP (red). Scale bar = 20 µm. Note the different magnification demonstrated by the scale bar. White arrow heads point to podocytes positive for the stained protein, respectively.
